# Urban and architectural risk factors for malaria in indigenous Amazonian settlements in Brazil: a typological analysis

**DOI:** 10.1186/s12936-015-0806-0

**Published:** 2015-07-22

**Authors:** Patricia Leandro-Reguillo, Richard Thomson-Luque, Wuelton M Monteiro, Marcus V G de Lacerda

**Affiliations:** Architect and Urban Planner, 13143 Thomasville Cr, Tampa, FL 33617 USA; Department of Global Health and Infectious Diseases, College of Public Health, University of South Florida, Tampa, FL USA; Fundação de Medicina Tropical Dr. Heitor Vieira Dourado, Av. Pedro Teixeira, 25, Dom Pedro, Manaus, AM 69040-000 Brazil; Universidade do Estado do Amazonas, Av. Pedro Teixeira, 25, Dom Pedro, Manaus, AM 69040-000 Brazil; Instituto de Pesquisas Leônidas and Maria Deane, Fundação Oswaldo Cruz, Rua Terezina, 476, Adrianópolis, Manaus, AM 69057-070 Brazil

**Keywords:** Malaria, Indigenous populations, Vector control, Architecture, Amazon

## Abstract

In the Amazon, m alaria is highly endemic in indigenous populations, which are often considered one of the last barriers to malaria elimination due to geographic isolation. Although the improvement of housing conditions is a good strategy towards the control and prevention of vector-borne diseases, such as malaria, this preventive practice has been barely undertaken in Latin America. An analysis of the architectural and urban features of indigenous Amazonian populations is essential to define and adapt these vector control measures. A total of 32 villages of 29 different ethnicities were studied and mapped by reviewing literature and visual information, and using a geographic information system. The most important architectural and urban characteristics influencing malaria were analysed according to the following categories: number of households and dimensions, supporting area, openings, materials, lifespan and location. Housing typologies found were classified within each of these variables. The results of this typological analysis included an easy-to-handle working template and revealing of features that benefit or hamper the presence of malaria vectors in Amerindians communities. Among risk factors, presence of open eaves, permeable walls, open-side constructions, large number of sleepers indoors, temporary-ephemeral houses, linear villages along stream banks, houseboats villages, poor urban drainage and villages surrounded by anthropogenic environments were highlighted. Indigenous settlements very permissive for anophelines were identified in ethnic groups, such as the Yanomami, Palikur, Paumari, Waimiri-Atroari and Wajãpi. Positive features were also recognized, including opaque and closed houses, large radial villages on bare soil, highly elevated stilted houses and the fire indoors, found among the Yawalapiti, Ashaninka, and Gavião-Parkatejê tribes. However, as Amazonian indigenous settlement typologies vary greatly even among villages of the same ethnic group, it is imperative to undertake an individual study for each community. Using the working template in Amazonian settlements it is possible to obtain data that will help researchers not only understand how architectural and urban features affect transmission, but also define vector control measures easily applicable by health authorities and acceptable by these communities.

## Background

Approximately 40% of the South American continent consists of the Amazon rainforest, of which 60% is located in northern Brazil [1]. In the Brazilian Legal Amazon, a socio-economical division composed by nine Amazon states, the population of Amerindians is approximately 433,363 people distributed into 226 ethnic groups [2]. Indigenous populations register disproportionately high indicators of poor health with high levels of maternal and infant mortality, malnutrition, cardiovascular illnesses, and infectious diseases, such as tuberculosis [3] and HIV/AIDS. Among them, vector-borne diseases are known to cause a significant burden in Amerindian communities of the Amazon basin, particularly malaria [4, 5]. The most favourable climate patterns for its propagation are mostly connected with high temperatures and humidity, which favour the survival of malaria vectors [6].

*Anopheles darlingi*, the main human malaria vector in Brazil, is present in about 80% of the country, however 99.8% of malaria cases are located in the Amazon Region [7]. This vector feeds all night with one, two, or three peaks of biting periods, being unimodal around midnight or bimodal with crepuscular rhythm. Although *A. darlingi* is more endophilic, endophagic and anthropophilic than any other *Anophele*s in the Americas, variations in activity, resting and biting patterns have been found thorough its distribution, with exophagic, exophilic and zoophilic behaviours reported, possibly caused by the use of insecticides [8, 9]. *Anopheles darlingi* usually bites inside dwellings and rests on interior walls no farther than one metre from the floor and on the ceiling, except in homes that have been treated with DDT, in which case it rests outside on vegetation [10]. Although it breeds in shaded margins of forested fluvial areas (rivers, streams, lakes, lagoons, ground pools), human landscape modifications result in an increasing number of breeding sites which are closer to homes. New ideal scenarios for larvae are pastures, flooded fields, irrigation canals, fish farms, wells and margins of roads [11, 12]. Adult *A. darlingi* often cluster in relatively small areas close to humans, mostly at ground level, not far from breeding sites and with a peak number occurring at the end of the rainy season, however, its flight range is greater than other sylvatic Anophelines, increasing its transmission potential [13].

**Figure 1:**
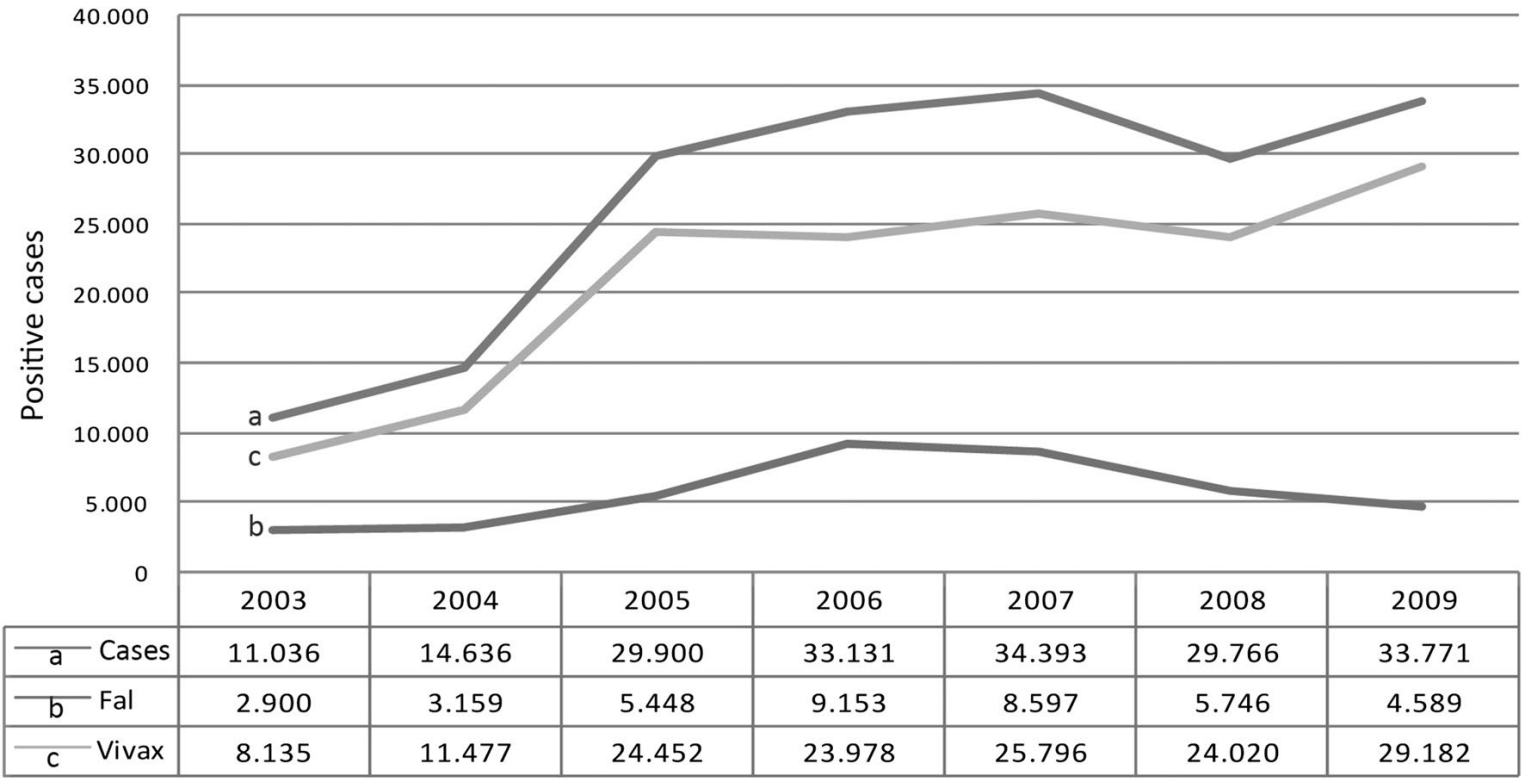
Malaria cases reported in the indigenous population of the Legal Amazon Region, 2003–2009. Source: Malaria-SIVEP (Information System of Epidemiological Surveillance). Updated 6.12.2010. FUNASA Management Report 2010. Health National Foundation, Health Ministry, Brasilia, Brazil, March 2011. p 129.

*Plasmodium falciparum* was introduced into South America by the transatlantic slave trade from Africa, after European colonization [14], and scientists have dated the introduction of *Plasmodium vivax* as coming via Melanesia and the Pacific several 1,000 years ago [15]. However, once Europeans arrived, Amerindians are presumed to have suddenly been exposed to a huge influx of new strains [16]. Development programmes in Brazil starting from the latter half of the 19th century prompted migration flows of white populations to the Amazon basin, triggering malaria epidemics. Since the 1970s, health authorities became concerned about the alarming decline in indigenous populations and scientific research confirmed that one of the predominant causes of mortality was *P. falciparum* malaria [17]. The renewed malaria control efforts have been successful, resulting in a 50% decrease of malaria cases in Brazil from 2000 to 2012 [18], particularly *P.falciparum* malaria, however this is not the case for indigenous populations. In 2001, 13,313 malaria cases were recorded in a population of 358,493 indigenous inhabitants representing an Annual Parasitic Incidence (API) of 37.1 malaria cases per thousand population, well above the average Amazon region (18.8) [19]. The number of Amerindians affected increased more than 300% from 2003 to 2009, especially for cases of *P. vivax* malaria (Figure 1). Therefore, Amazon indigenous communities can be considered as residual reservoirs for malaria infection in the majority of the areas where this disease is being highly controlled in Brazil. In Mesoamerican indigenous population the situation is not different, and it poses a great challenge for malaria elimination in that area [20].

Dwelling modifications are a good ally in vector borne disease control and elimination [21–23], and house improvements such as screened windows, screened ceilings [24], closed eaves [25] or raised floors [26] are commonly suggested in the literature. The enormous potential of architecture should be considered as a helpful tool in the great challenge of malaria elimination. Nevertheless architectural preventive practices have been barely undertaken in Latin America, consisting mainly of local screened solutions in emergency situations, such as the Oswaldo Cruz’s screened windows for malaria elimination in the Madeira Mamoré Railway construction in 1910 in Brazil [27], or the yellow fever-malaria portable Quarantine Station created by William Gorgas in 1905 in the Panama Canal [28].

The urban spaces generated between constructions and the characteristics of the settlement’s surrounding perimeter are also critical for malaria control. In 1940, the newly formed Sanitation Commission of the Amazon in Brazil conducted one of the first studies on the incidence of malaria in cities situated on the Amazon banks and their major tributaries. Those surveys were based on the conditions of occurrence of the disease, including characteristics of households, environmental characteristics of the peri-domestic area, outbreaks, and finally mapping of endemic areas [29]. However, this plan did not cover indigenous villages.

### Indigenous Amazonian settlements today

Indigenous Amazonian settlements’ features have suffered constant transformations and some traditional households typologies have disappeared [30]. Yet they can be considered the result of centuries of progressive environmental adaptation, and represent the physical entity where culture and its expressions are practiced.

One of the first main physical modifications made by whites was through Salesians missions in the early 20th century, adding new constructions for Catholic rituals and boarding schools, and destroying collective “malocas”, which were replaced with single family homes under the pretext of promiscuity and lack of hygiene [31]. Recently, Evangelical missions have caused an increase in the population concentration and sedentarization among indigenous settlements [32]. These changes occurred due to a higher number of households and the use of new materials and construction techniques that prolong lifespan which have favoured desertion of small and remote villages. In many of these villages, the use of the traditional “maloca” has changed from housing-collective to ritual-religious use.

Since the late 20th century, various government agencies and non-governmental organizations (NGOs) have been implementing improvements in sanitation, drinking water infrastructure, projects for dwelling renovations and new housing typologies in settled indigenous villages [33]. Furthermore, many Amerindian communities, especially those localized in the vicinity of large non-indigenous populations, have been progressively adopting the urban and housing typology of regional villages.

Today, an enhancement of cultural, territorial and architectural heritage of indigenous Amazonian ethnicities is witnessed. Any intervention in these settings requires prior ethnographic and anthropological assessments. In addition to such appraisals, an analysis and classification of architectural and urban features of these settlements is also needed if we want to define and adapt malaria vector control measures in Amerindian populations in a context where environment, culture and architecture go hand in hand.

## Methods

Literature and visual information (still photography and video recording) was reviewed on indigenous settlements in the Amazon rainforest mostly from the Brazilian Amazon basin, a vast territory that comprises a high number of Amerindian settlements and a great diversity of cultures and tribes (estimated 226 of the 240 indigenous ethnicities located in Brazil) [2]. Search tools such as PubMed, SciELO Brazil and Google for the following terms were used: “Brazil”, “indigenous”, “malaria”, “architecture”, “urban”, “anthropology”, “ethnography”, “households” and “villages”. The retrieved information allowed gathering a large number of publications and studies on the tribes that inhabit the Brazilian Amazon basin [30–47].

**Figure 2:**
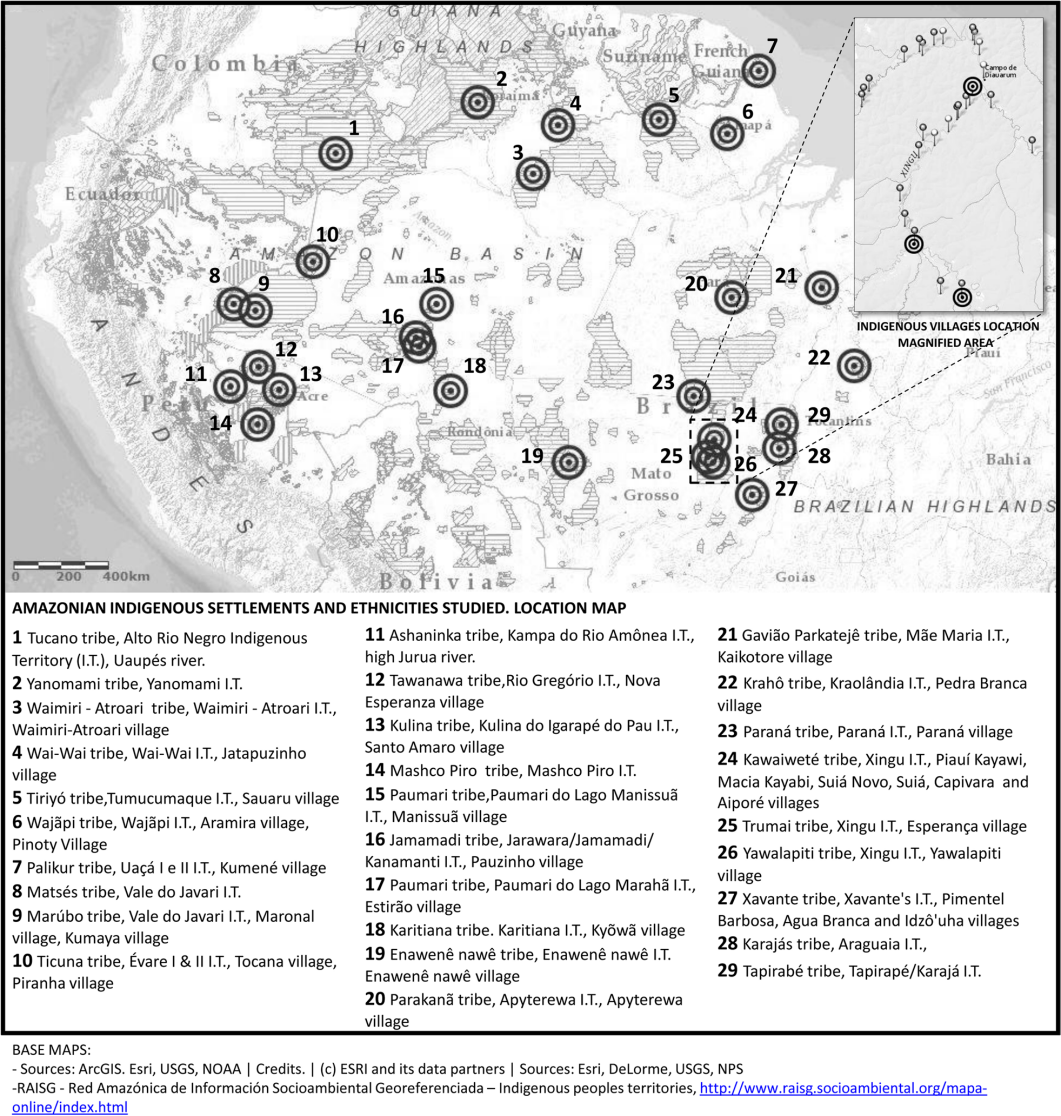
Location map of the Amazonian indigenous settlements and ethnicities studied.

Books and articles written by professionals in architecture and urbanism provided a general basis for a first classification of settlements. However, an elevated proportion of dwellings described were already extinct and authors focused on the most representative and solid constructions, ignoring mixed-influenced and ephemeral households. Ethnographic and anthropological works carried out on ethnic groups and indigenous territories provided a wide range of alternative indigenous settlements that increased the list of ethnicities and villages studied. The use of a Geographic Information System (ArcGIS online) helped to map and locate many of these settlements in their Indigenous Territories, legally protected areas inhabited and owned by indigenous people (Figure 2).

Although the number of tribes studied was not very high compared with the existing ones in the Legal Amazon they represent the wide variety of indigenous Amazonian settlements. Many indigenous ethnicities share the same constructive and urban typology—particularly if located in similar climate and environmental conditions—as in the Upper and Middle Rio Negro or in the Xingu River, and numerous communities have been gradually adopting regional typologies.

**Figure 3:**
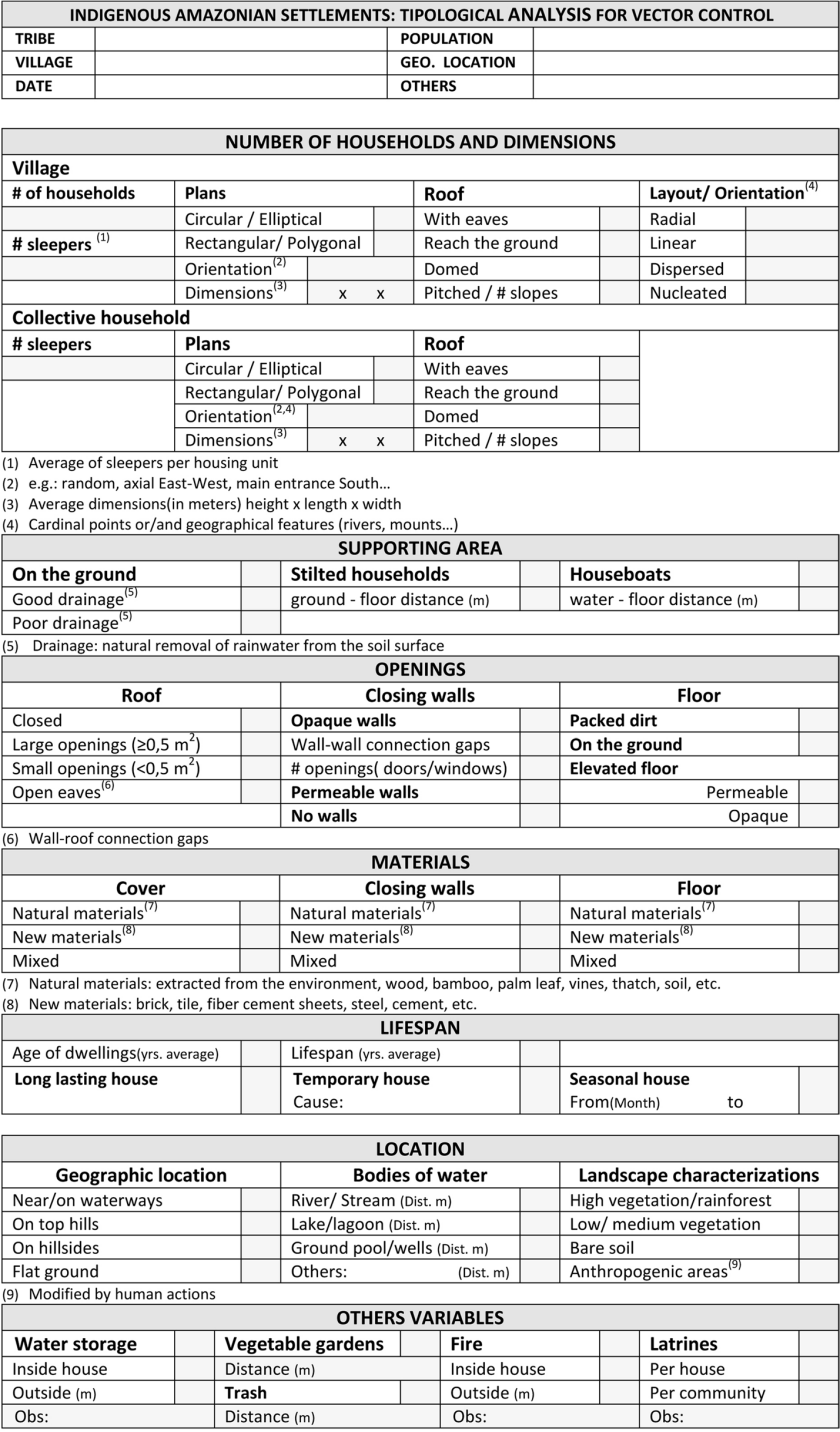
Working template for on-site study of Amazon indigenous population’s dwellings.

The most important architectural and urban factors influencing malaria in indigenous Amazonian settlements were standardized into different categories, which were also used to shape a working template for on-site study (Figure 3): (1) number of households and dimensions, (2) supporting area, (3) openings, (4) materials, (5) lifespan and (6) location. For each of these variables the diverse features found were sub-classified, and distinguished those that benefit from those hampering the presence of malaria vectors in Amerindian communities.

This approach may differ from other studies that mainly focus on the description of the housing model as a whole [48, 49]. The purpose of this was to simplify the identification of malaria risk factors in statistical analysis in indigenous communities.

## Results

### Number of households and dimensions

Certain malaria epidemiological studies noticed how factors related to household architecture, such as number of sleepers or room/house dimensions, were introduced as possibly influential on malaria incidence [50–52]. However, other urban design features have not been taken into consideration such as houses’ proximity, disposition and orientation, which are paramount factors affecting public health issues such as ventilation, lighting and thermal comfort [53].

**Figure 4:**
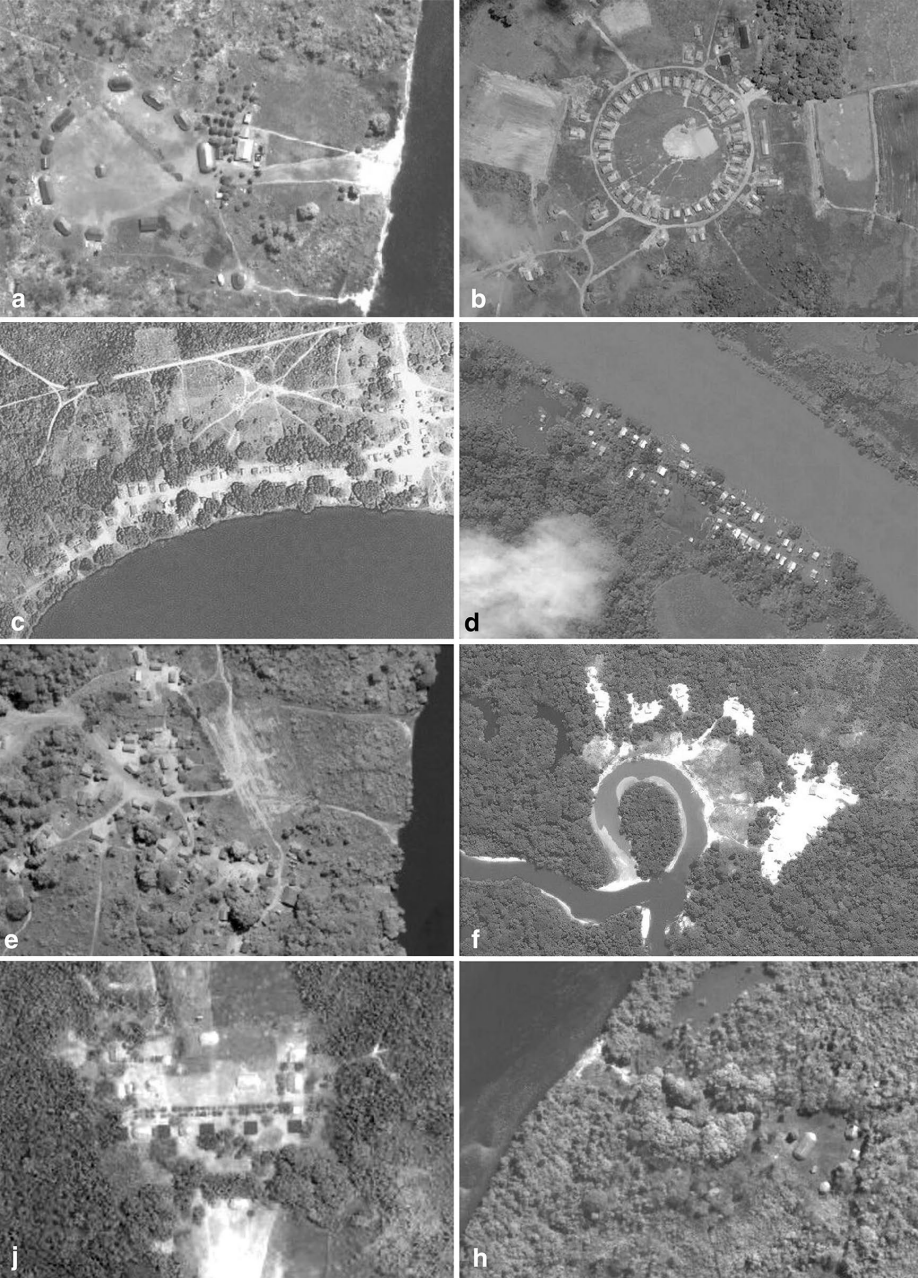
Indigenous Amazonian settlements, location and disposition. **a** Radial disposition, Esperança village, Trumai tribe. **b** Radial disposition, Kaikotore village, Gavião Parkatejê tribe. **c** Lineal disposition, Karajás tribe village. **d** Lineal disposition, Tocana village, Ticuna tribe. **e** Dispersed disposition, Tapirabé tribe village. **f** Dispersed disposition, Piranha village, Ticuna tribe. **g** Nucleated disposition, Aiporé village, Kawaiweté tribe. **h** Nucleated disposition, Suiá Novo village, Kawaiweté tribe. Source: ArcGIS Online.

Settlements were classified as villages or collective households. Villages are composed of different homes that vary in number according to the existing families. House plans can be circular, elliptical, rectangular or polygonal. Coverage ranges from domed to pitched roofs, and the latter may present one, two or up to four slopes. Covers can even reach the ground, removing eaves. In the case of “ocas” and “malocas”, these traditional households can accommodate several families linked by kinship relations. According to their layout, villages can be radial, linear, dispersed or nucleated (Figure 4), mainly oriented following geographical features, such as rivers or mounds, but also cardinal directions.

**Figure 5:**
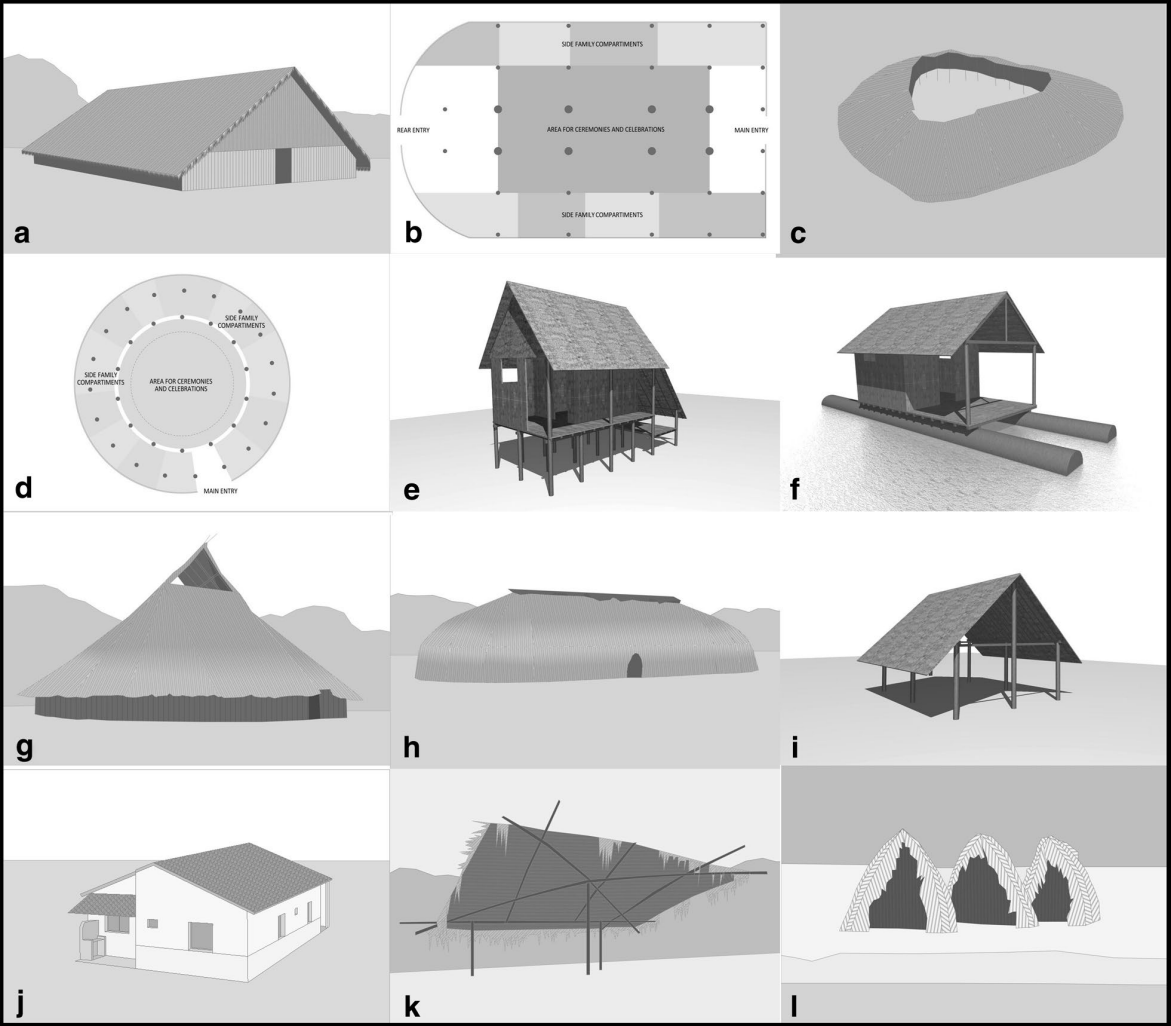
Indigenous Amazonian settlements, typologies and features. **a** Traditional maloca of upper and lower Negro river. **b** Maloca, use and distribution, floor plan. **c** Collective households: Yanomami’s shabono. **d** Yanomami’s shabono, use and distribution, floor plan. **e** Stilted households: Ashaninka tribe’s housing in high Juruá River. **f** Houseboats: Estirão village houseboat in Marahã lake, Paumari tribe. **g** Cover with small openings: Vaupes’ maloca. **h** Closed cover: Yawalapiti village, Xinguana tribe. **i** Without walls: Jamamadi tribe’s housing in Pauzinho village. **j** New materials and constructive techniques: Gavião Parkatejê tribe, Kaikotore village housing. **k** Temporary dwelling: the tapiri, Yanomami tribe. **l** Temporary dwelling for dry season, Mashco-piro Tribe.

Collective households are the simplest form of organization. The whole tribe lives under the same roof. The “maloca”, used in these cases as a collective household, is divided into several open-side compartments, each inhabited by a nuclear family. During the celebrations and especially in the more formal ceremonies, the space is rearranged and the center of the residence becomes the most important area, where the dance takes place. Floor plans are generally rectangular with a short sidewall or both semicircular (Figure 5a, b). The Yanomami’s collective household, the “shabono”, is circular or polygonal with a square in the center, each side of the polygon corresponding to a family residence. Its size is proportional the number of people it houses, some being able to accommodate up to 400 inhabitants. The lightweight covers of housing units are usually hinged to form a single surface that offers complete shelter, forming a truncated cone with the top open for sunlight and smoke outlet (Figure 5c, d).

Large number of confined sleepers facilitates transmission since several people can be infected by the same mosquito. Linear villages along stream banks or nucleated villages located near breeding sites are also risk features as their urban layouts in relation to aquatic environments favor the presence of malaria vectors throughout the settlement. Features such as the absence of corners, large interior dimensions (including height), low urban density and dominant wind orientation, have not yet been thoroughly studied in malaria epidemiological studies. These features may result in a reduction in the number of mosquitoes both inside and outside households. Finally, radial settlements, common among Xingu River’s ethnicities, are raised upon bare soil with a large diameter, favoring airflow and evaporation, beneficial for vector prevention in peri-domestic areas.

### Supporting area

The benefits to humans that elevated constructions generate in relation to vector behavior have been reported [26, 54]. On the other hand, the close relationship between malaria and proximity of homes to water-bodies is critical in these settings [55], especially for communities that live on the water surface.

Amerindian houses are commonly built on the ground where a good drainage is essential as floors are typically packed dirt. Other typologies adapted to watercourses and their floods were found. Stilted households are separated from the ground and supported by simple wooden stakes that can last for nearly 100 years. They are made of boards, slightly apart to allow air circulation, and can either have light walls forming one or two differentiated areas, or no walls. The distance between the ground and the elevated floor can range from 60 cm to 2 m (Figure 5e). Houseboats, which often have the same characteristics as indigenous stilted dwellings, have thick logs that float on the water for more than 50 years (Figure 5f). They are considered the Paumari tribe’s typical house, however today they represent a minority, as they are difficult to remove and remain tied for long periods at the edge of lakes or rivers, following only the changes in water levels. This type of dwelling does not prevent its residents from exercising their activities on land. Thus, houseboat typology and poor urban drainage are recognized as features that stimulate the increase of malaria vectors in indigenous communities, while highly elevated stilted houses typology lessen the number of mosquitoes indoors.

### Openings

Openings in dwellings have been a recurrent risk factor in malaria vector-control studies related to typologies, mainly there are three house-entry areas: eaves, walls openings and roofs openings [50, 52, 56, 57].

Coverage is an essential building element and even more in tropical or equatorial climates, where it provides protection from the sun and the rain. Among the indigenous constructions studied, most featured small openings in roofs that generally operate as smoke outlets, allowing lighting and ventilation (Figure 5g). Other dwellings presented closed covers, continuous on all sides and without perforations, or covers with large openings, as the aforementioned Yanomami’s “shabono”, where the opening coincides with the central area (Figure 5d). In certain indigenous households the roof reaches the ground for rain and wind protection, eliminating eaves (Figure 5h).

**Figure 6:**
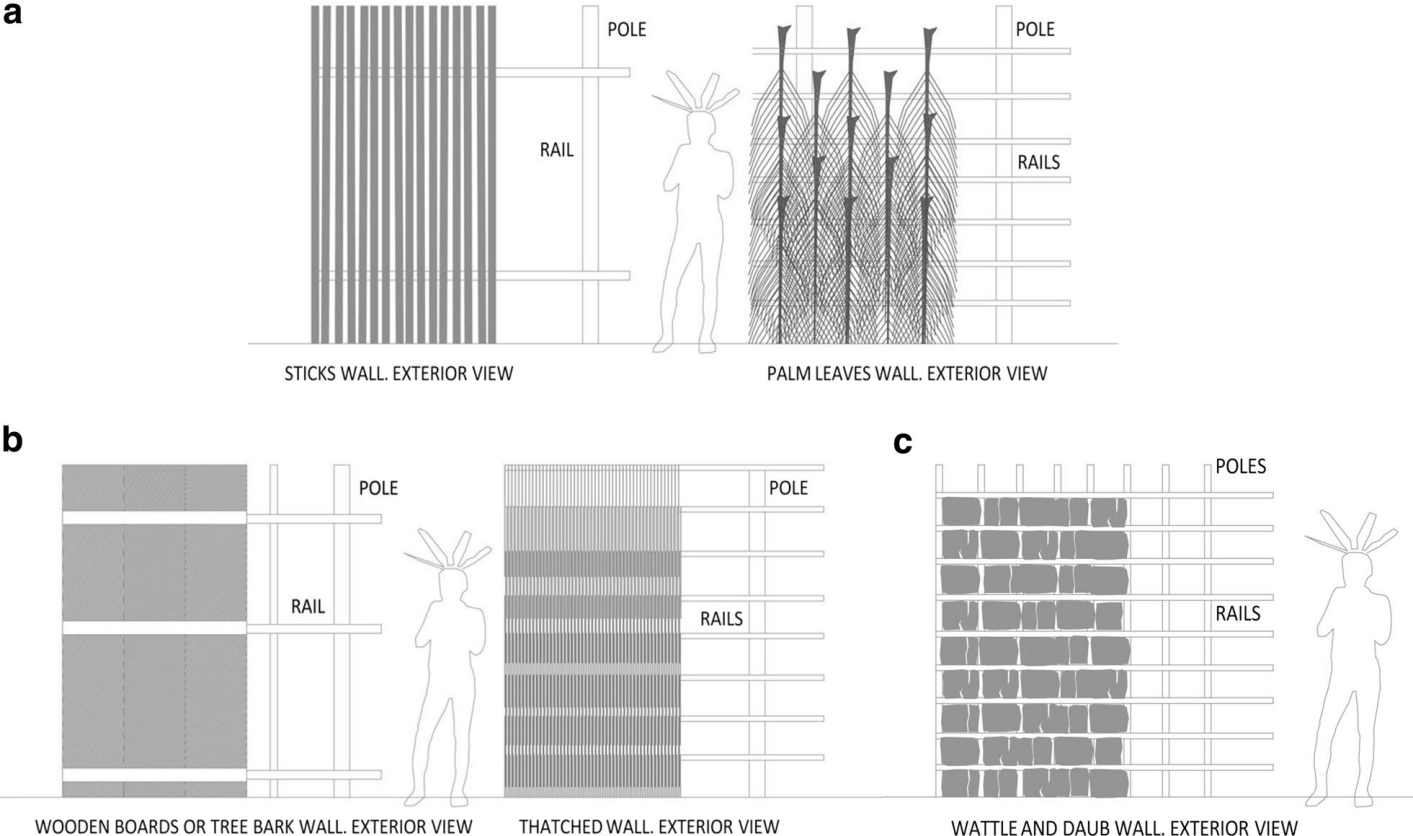
Walls openings. **a** Permeable walls examples: sticks and palm leaves walls. **b** Opaque walls examples: wooden or bark boards and thatched walls. **c** Wattle and daub constructive technique.

A high diversity of materials and designs for indigenous houses’ walls were observed, resulting in a wide permeability range as defined by interstitial spaces that allow natural ventilation and certain grade of lighting inside houses (Figure 6a). Some other walls were opaque, made without perforations except those required for access, lighting and ventilation (windows and doors) (Figure 6b), and thus, it is necessary pay special attention to wall-to-wall and wall-to-roof connections as gaps for mosquito entry. Some communities located in a hot and humid equatorial climate raised open side structures, without any closure, ensuring air circulation and moisture removal (Figure 5i). In warmer climates further South there was a progressive closing of constructions. Floors in stilted households and houseboats typologies, separated from ground or water surfaces, could also be permeable or opaque.

Covers with large openings, open eaves, permeable walls and floors and open side households are constructive characteristics that favour entry of malaria vectors. Thus, the more opaque and closed a house is, the more likely the presence of mosquitoes inside is reduced.

### Materials

Housing materials have been considered when evaluating malaria incidence inside households, and natural materials have been usually connected to an elevated number of *Anopheles* mosquitoes indoors [58]. The presence of palm fronds or mud walls in construction attracts pest infestation, as is the case for the vector of Chagas disease [59]. However, in some epidemiological studies the use of natural materials in housing is usually wrongly linked with poor construction, meanwhile the utilization of new materials is commonly associated with a good and healthy house [51]. Many Amerindians’ traditional constructions are complex and strong structures, environmentally adapted and with high thermal comfort.

Generally, the construction techniques and materials used were similar among tribes. Natural materials extracted from nearby surroundings such as wood, bamboo, palm leaf, vines, and thatch, are traditionally used. However, in some cases they have been gradually replaced by newer building materials, such as brick, fiber-cement sheet, steel, and cement. For example, the Kaikotore village, erected in 1984 for the Gavião Parkatejê tribe and designed in a radial disposition, is composed of 33 brick and tiled roof houses equipped with water, electricity and drainage systems (Figures 4b, 5j) [33]. The wattle-and-daub construction technique has also been adopted, consisting of interweaving vertical timbers set in the ground with horizontal beams, rising into a large perforated panel that after having filled the gaps with clay was transformed into a wall (Figure 6c).

Natural materials possess low heat capacity, favouring thermal comfort inside households. The utilization of new materials, mostly with high heat capacity, could deteriorate this comfort if the house is not well designed and ventilation is not guaranteed.

### Lifespan

Very few studies have introduced the age of houses as a parameter that may influence malaria transmission, yielding inconclusive results [60]. Lifespan in indigenous structures has not traditionally been very long as a result of several factors including depletion of hunting and other resources nearby, quick deterioration due to the nature of materials and its location within the Tropical and Equatorial forest, invasive pests, such as cockroaches or spiders, internal conflicts, disease outbreaks, and deaths. The useful life of constructions ranges from two to 30 years, the village or house is then burned or abandoned to build another on the same site or in a different area. Besides these primary constructions, temporary-ephemeral dwellings are also commonly erected to live in meanwhile a new village is built or to purchase food and materials far away from the original settlement (Figure 5k, l). Seasonal houses are also commonly used during rainy or dry seasons, and are occupied depending on river floods. However due to the previously described phenomena of concentration and sedentarization of this population, lifespan of households has increased significantly. In some cases they become nearly permanent as a consequence of the use of new building materials, new urbanization and sanitation projects and installation of evangelical missions [32]. Therefore, temporary-ephemeral households, with light weight structures and incomplete, are identified as a housing typology conducive to malaria vector entry.

### Location

Geographic location and landscape characterizations of a settlement are important factors affecting malaria incidence [61]. Although the Amazon region is considered a whole year transmission area, malaria transmission increases significantly during the rainy season and is strongly associated with watercourses proximity [62, 63]. As Amerindians usually need rivers or streams for subsistence, houses are commonly raised in their vicinity, on riverbanks, or on watercourse surfaces. Other geographic locations are on top of a hill, on hillsides or in flat ground, both in rainforest and deforested areas (Figure 4).

Putative risk factors for higher malaria incidence would be villages set on stream banks, houseboat villages and villages surrounded by anthropogenic environments (land transformed by human action).

### Other factors

Not as strongly linked to the indigenous settlements’ architecture but decisive for malaria control in these populations is the effect of fire inside the houses, which reduces the presence of mosquitoes indoors although it is a health hazard [64]. Existence of water storage, vegetable gardens and trash accumulation areas located near dwellings increase the risk of malaria in the population [50, 53, 65]. As indigenous households have adapted progressively to Amazonian regional constructions, the presence of latrines, septic tanks and others sanitation facilities has increased, therefore an analysis of their features and sanitation conditions to evaluate hazards is recommended. Table 1 summarizes the assessment of urban and architectural features in indigenous Amazonian settlements likely favouring malaria transmission.

**Table 1:** Urban and architectural features in indigenous Amazonian settlements likely favouring malaria transmission

Urban and architectural risk factors for malaria in indigenous Amazonian settlements		
Category	Risk factors	Description
Number of households and dimensions	Collective household Nucleated village	Confined dwellers favor the spreading of infective mosquitoes and increase the chances of uninfected mosquitoes to acquire the human infective sexual stages
Supporting area	Houseboat Poor urban drainage	Establishment of breeding sites for *Anopheles* mosquitoes close to houses, increasing man-vector contact
Openings	Cover with large openings Open eaves Permeable walls and floors open-side household	Houses become permissive for *Anopheles* mosquitoes entry, increasing man-vector contact, and ineffectiveness of vector control measures
Materials	Natural materials	These materials promotes high number of indoor resting malaria vectors, increasing man-vector contact
Lifespan	Temporary-ephemeral household	Light-weight and incomplete houses conducive to stimulate malaria vector entry
Location	Village on stream bank Water village Village in anthropogenic area	Landscapes modified by man action and water courses are propitious environments for *Anopheles* breeding sites, nearby villages are conducive for man-vector contact
Other	Water storage/vegetable gardens/trash areas near household Poor state of sanitation facilities	High attractiveness of *Anopheles* mosquitoes to settlements and establishment of resting sites close to houses, increasing man-vector contact

## Discussion

In several of the early writings from the 16th century conquerors describe Amerindians populations as “very healthy”. Some Jesuits missions reported that they did “not have knowledge of people dying of fever but only of old age”, and they used to “live up to their 90s” [66]. Recent Amazon archaeology findings revealed the existence of pre-Colombian indigenous societies who lived in large villages, or village networks connected with roads, that could host thousands of inhabitants. They possessed large crop fields, orchards and fish farms that became obsolete after overseas epidemics decimated their population [67–69].

Structural characteristics of settlements are historical legacies that allow to understand the needs of comfort and use of their inhabitants. Despite the great diversity in typologies and characteristics among indigenous Amazonian settlements, some of their common features such as large coverage and permeable structures, show that environment protection and climate adaptation are the most important challenges. However, these constructions have historically suffered alterations due to new needs and external influences.

It was not observed a close relationship between the proximity of indigenous Amazonian households to watercourses and the opacity of their enclosure. However, literature described extinct types of houses directly related to the Amazonian watercourses as designed to prevent the access of insects. In the former Paumari’s houseboats anchored in the Purus River, where fire was located on the banks and houseboats were mainly used for sleeping, constructions had a single entrance covered by a mat that protected from heat and insects [32]. Another example are the extinct Ticunas’ “malocas” near the border with Peru. Two types of “malocas” were described: open-sided, located far from the watercourse, and closed, settled near the river for protection from mosquitoes [70]. Current “malocas” are of the closed type, ensuring protection from attacks, as an adaptation due mostly to periods of conflict experienced by its inhabitants.

Malaria studies carried out in different indigenous villages such as Xavánte’s [71] and Waimirí-Atroari’s [72] also suggest that indigenous households’ architecture could act as protection against vector entry, pointing to factors such as opacity of their enclosures, minimum openings, darkness and especially the smoke effect from fires. Fire may be the most common preventive measure used by indigenous people for vector control. In most villages, it remains lit overnight, and is located in sleeping areas. It produces a protective tar layer on walls and roof that may reduce insect’s presence indoors. The traditional and almost abandoned technique of using smoke for housing materials preservation makes households more durable and protects them from plagues and fungus [73]. Revised smoke preservation techniques could be a valuable tool in fighting malaria among Amerindians.

However, the fire indoors, along with thermal discomfort, are factors that inhibit the acceptability and compliance with the use of impregnated bed nets among Amerindians, which are among the best practices for malaria control. This is critical in communities in which many families sleep under the same shelter, enabling possible malaria-infected mosquitoes to feed from multiple sources and spread disease. This would favour the spread of infective mosquitoes and increase the chances of uninfected mosquitoes to acquire the *Plasmodium* parasite from humans.

*Anopheles darlingi* behaviour may also differ between typologically different Amerindian settlements. Research on malaria vectors conducted in two indigenous villages located in the northern Brazilian state of Pará showed how the number of *A. darlingi* collected in a Zo’é community was more than 10 times greater than in a Wai–Wai village. The mosquitoes from the Zo’é community showed a considerably more active pattern, a behaviour attributed to different characteristics of the constructions and the way of life of ethnic groups. The first settlement had semi-nomadic habits, was smaller and located in the forest, with households consisting of straw-roofed without walls. The second village was close to the river and dwellings were made of natural materials, with walls without windows and a single entrance; the number of mosquitoes collected indoors was less than 10% of the total [74]. Nevertheless, it will be necessary to conduct more research on these aspects in order to clarify connections between indigenous households and vectors.

Amazon native populations used to live in symbiosis with nature. Non-native people’s influence, water pollution, and the forced displacement of populations other than those traditionally inhabited areas, have brought major changes in these communities and severely affected their traditionally sustainable lifestyle. A striking feature of most indigenous areas was the precarious sanitation conditions. Villages often lack waste collection and drinking water infrastructure, and although some improvements have been implemented in certain settled Amerindian communities since the late 20th century, they did not guarantee public health amelioration due to bad designs and absence of maintenance, promoting breeding sites for *A. darlingi*. Moreover, certain studies undertaken in settlements with vegetable farms nearby have demonstrated that these communities are permanently exposed to malaria throughout the year [75]. As indigenous people farm much of their food base in orchards, the proximity of these to settlements is also relevant.

As previously highlighted, urban and house improvement implementations are helpful tools for malaria prevention. To ensure effectiveness of these improvements among Amerindian communities is essential to be respectful of these populations’ way of life and easy to implement, without interfering, damaging or modifying their culture, environment or thermal comfort inside constructions. However, there are many challenges to be faced such as open-side constructions, short lifespan, and ephemerality. In these cases, the versatility and adaptability of protective measures proposed are of paramount importance.

Malaria is, and has been for several centuries, an important health problem in Amazon Amerindians. The implementation of an information system on indigenous health is of vital importance, and control programmes should be adapted to particular characteristics of indigenous areas. The use of the easy-to-handle template proposed here could provide useful information about protective and risk factors in each indigenous Amazonian settlement and would help to identify suitable urban and architectural improvements to reduce malaria cases among inhabitants.

Cultural and social characteristics of tribes must be taken into account and each community should actively participate in the whole process, from data collection to final implementation, including helping in the design. When possible, measures should be environmentally friendly and make use of local and natural materials. Information, training and awareness initiatives will also be necessary to ensure proper use and maintenance of measures proposed.

Among household improvement actions we suggest to encourage the use of stilted constructions and opaque walls as well as high-rise and ventilated covers, screens on windows and ventilations gaps, and the removal of open-eaves. Urban improvement actions would include drainage amelioration, better orientation and disposition of houses to facilitate ventilation and evaporation, improvement or implementation of water supply and sanitation systems, waste management and village cleaning actions, relocation of orchards, and stagnant water elimination.

## Conclusions

Typologies of indigenous Amazon settlements vary greatly depending on numerous factors, such as way of life, tribal culture, climate, type of environment, materials available for each community and non-native influence. It is noteworthy that even among villages of the same ethnic group the characteristics of their communities may differ significantly. Efforts in controlling and eliminating malaria among these populations face several challenges that are difficult to resolve. In this context, urban and house improvement implementations, as a measure for vector control, may be useful. Unfortunately, due to great architectural and urban differences among settlements, this cannot be accomplished under a unified approach, and studies must be tailored to specific villages. The use of the proposed working template would be useful for understanding how architectural and urban features affect malaria transmission in each settlement, and in helping to design malaria prevention measures, including house and urban improvement actions.
